# Technological Solutions to Improve Inpatient Handover in the Era of Artificial Intelligence: Scoping Review

**DOI:** 10.2196/70358

**Published:** 2025-07-31

**Authors:** Louis Agha-Mir-Salim, Isabelle Rose Alberto, Nicole Rose Alberto, Leo Anthony Celi, Pia Gabrielle Alfonso, Rachel Hicklen, Katelyn Legaspi, Rajiv Hans Menghrajani, Faye Yu Ng, Patricia Therese Pile, Christopher M Sauer

**Affiliations:** 1 Institute of Medical Informatics, Charité – Universitätsmedizin Berlin, Corporate Member of Freie Universität Berlin and Humboldt-Universität zu Berlin, Berlin, Germany; 2 College of Medicine, University of the Philippines Manila, Manila, Philippines; 3 Department of Obstetrics and Gynecology, Philippine General Hospital, Manila, Philippines; 4 Laboratory for Computational Physiology, Massachusetts Institute of Technology, Cambridge, MA, United States; 5 Library, The University of Texas MD Anderson Cancer Center, Houston, TX, United States; 6 Department of Medicine, Lincoln Medical Center, New York, NY, United States; 7 Yong Loo Lin School of Medicine, National University of Singapore, Singapore, Singapore; 8 Department of Medicine, UConn Health, Farmington, CT, United States; 9 Institute for AI in Medicine, Laboratory for Clinical Research and Real-World Evidence, Essen University Hospital, Hufelandstr. 55, Essen, 45147, Germany, +49 201 723 0; 10 Department of Hematology and Stem Cell Transplantation, Laboratory for Clinical Research and Real-World Evidence, Essen University Hospital, Essen, Germany

**Keywords:** clinical handover, workflow improvement, technological solution, SBAR, I-PASS, artificial intelligence, AI, (I)SBAR

## Abstract

**Background:**

Clinical care globally faces increasing strain due to escalating documentation demands. Simultaneously, technological solutions for clinical workflows, particularly inpatient handovers, are being developed to alleviate workforce stress. However, the maturity, adoption scale, and impact of these technologies on clinical practice remain unclear.

**Objective:**

To address this gap, we conducted a scoping review to summarize current advancements in technological solutions for inpatient handovers.

**Methods:**

This study was prospectively registered on Open Science Framework. Publications from January 1, 2010, to January 1, 2024, were retrieved from MEDLINE, Embase, Cochrane Library, and Scopus. To be included in this review, studies were required to focus on (1) the implementation, assessment, or enhancement of health care provider handover workflows; (2) inpatient setting; and (3) the proposal or implementation of one or more technological solutions. Abstract and full-text screenings were conducted independently by 2 reviewers, with conflicts resolved by a third reviewer. Data extraction and synthesis were performed by multiple authors and cross-reviewed for accuracy.

**Results:**

The search identified 779 publications, of which 53 met the inclusion criteria. Analysis revealed a predominance of low-complexity technologies, such as electronic checklists, with limited exploration of advanced solutions like natural language processing. Most studies were in the pilot stage (33/53, 62%), while some described documented implementations (11/53, 21%). Reported outcomes included improvements in the completeness, accuracy, and consistency of critical information during patient transfers (20/53, 38%). Challenges included scalability, inconsistent adoption, and difficulties integrating advanced technologies into existing workflows.

**Conclusions:**

Low-complexity technological solutions show potential for enhancing inpatient handovers but face barriers to scalability and sustained adoption. While artificial intelligence (AI) has the potential to bring transformative benefits, a limitation of this review is that none of the included studies reported successful clinical implementations of AI solutions aimed at improving handover processes.

## Introduction

Artificial intelligence (AI) and machine learning (ML) applications are increasingly implemented in clinical practice. However, postimplementation analyses showed unexpected or ambiguous results in clinical practice with regard to an improvement of clinical workflow or outcome. For instance, a recent study showed large heterogeneity as to which radiologists benefit from the use of AI assistance [1]. Interestingly, factors like years of experience, specialty, and prior use of AI tools did not reliably predict how a radiologist will be affected by AI assistance; that is, lower-performing radiologists at baseline did not consistently benefit from AI, with some improving, some worsening, and some seeing no change. This led us to ask whether, instead of implementing AI solutions into existing workflows, workflow analysis and optimization should precede AI development and be considered when implementing AI into clinical workflows. Workflow optimization has repeatedly been highlighted as a key priority to optimize health care delivery, improve patient safety, decrease strain on physicians, and lower burnout rates [2-4].

A frequent workflow in inpatient practice is patient handover. The British Medical Association and National Patient Safety Agency define handover as “the transfer of professional responsibility and accountability for some or all aspects of care for a patient, or group of patients, to another person or professional group on a temporary or permanent basis” [5]. The advent of multidisciplinary care and shift work, especially in the inpatient setting, requires a rotating cast of health professionals, each of whom needs to be apprised of the patient’s needs during a handover. The aim of the handover is to provide the patient with continuity of care by ensuring that the incoming team can adequately take charge of the patient’s care. However, each of these moments intended to ensure continuity is, in itself, a potential point of discontinuity. A poorly performed handover could lead to the loss of critical information, poor precision or efficacy, and maybe even outright error [6]. Poor handovers are associated with multiple potentially preventable hazards [7].

One of the most commonly used methods of improving handovers is the use of a standardization mnemonic [2]. The evidence for standardization has been conflicted. A systematic review [8] found that while there were multiple studies that showed increased positive outcomes, there were also studies that showed mixed, insignificant, and even negative outcomes for standardization. The mnemonics are meant to provide structure to the information reported during the handover. It limits the scope to what is relevant for the patient’s immediate care, omitting irrelevant data while preserving critical information. Popular examples of these standardization mnemonics include (I)SBAR (Introduction, Situation, Background, Assessment, Recommendation) [6] and I-PASS (Illness severity, Patient summary, Action items, Situation awareness and contingency planning, Synthesis by receiver) [9].

Despite these existing frameworks and guidelines for clinical handovers, omission of necessary details and inclusion of irrelevant points frequently occur [2]. Clinical inpatient handovers may therefore be an appealing use case to benefit from novel technologies, such as automated summaries using large language models (LLMs). On a larger scale, technological solutions have transformed work processes by reducing human errors, improving clinical outcomes, increasing practice efficiencies, reducing variation in practice, facilitating information sharing, and recording data over time [10]. One prominent example is the changes to clinical processes and systems that have occurred during and stayed beyond the COVID-19 pandemic [11]. Digital tools enabled hospitals to continue offering health services virtually through digitization of information, electronic booking and referrals, telemedicine, and virtual clinics [11,12]. These novel approaches to care delivery have the potential to be efficient, collaborative, cost-effective, and patient-centered [12].

To date, while technology has been proposed as an opportunity for redesigning clinician workflows [13], such as electronic health records [14], there remains a gap in understanding the specific technological solutions that have been proposed or implemented to improve inpatient handover. This includes which professional groups are involved, the settings in which handovers occur, and whether the impact on workflow and outcomes is positive or negative. In addition, there is limited knowledge about the rationale for using technology to optimize handovers, as well as the degree of technical maturity of the solutions used, which refers to a tool’s readiness for routine use, including the proposal of a model or solution, pilot implementation, or full integration into clinical workflows. Therefore, the purpose of our study was to systematically scope the current state of handover improvement studies that propose technological solutions. We sought to identify the settings in which handovers are being optimized, the care groups involved, the types of technologies used, and the advantages and disadvantages of these workflow strategies. Our goal was to report on best practices, advantages, and disadvantages, including the status quo of implemented solutions.

## Methods

### Protocol and Registration

The results of this scoping review are reported according to the PRISMA-ScR (Preferred Reporting Items for Systematic Reviews and Meta-Analyses extension for Scoping Reviews) guidelines [15] (the PRISMA-ScR checklist is provided in Checklist 1). The review protocol has been developed in accordance with the guidelines by the JBI (Joanna Briggs Institute) Scoping Review Methodology Group [16]. It was first registered a priori on February 22, 2024, on Open Science Framework and has been updated on March 19, 2025, to improve clarity and consistency with the manuscript [17].

### Eligibility Criteria

To define the eligibility criteria, we used the PCC (Population, Concept, and Context) framework [18]. The final eligibility criteria are listed in Table 1.

**Table 1.** Eligibility criteria following the PCC (Population, Concept, and Context) framework and additional restrictions^a^.

	Inclusion criteria	Exclusion criteria
Population	Inpatient encounters	All non-inpatient encounters (eg, interhospital care) and patient discharge
Concept	Technological solutions for clinician-to-clinician handover	Technological solutions that (1) are not central to handover, (2) depend on patient input, (3) are not explicitly technology based, or (4) only serve as a support mechanism
Context	Implementation, assessment, or enhancement of handover workflows between health care providers	Any other context

a Additional restrictions include (1) study design, encompassing primary studies (prospective and retrospective observational studies and interventional studies) and secondary studies; and (2) language, limited to papers published in English, German, or Filipino.

### Search Strategy

We conducted a literature search on January 18, 2024, of MEDLINE Ovid, Embase Ovid, Cochrane Library, and Scopus, including publications from January 1, 2010, to January 1, 2024. The search terms included were (1) workflow terms, (2) redesign terms, (3) provider terms, and (4) communication terms, including relevant MeSH (Medical Subject Headings) terms. The search strategy was developed iteratively with the help of an information retrieval specialist, with adjustments based on the sensitivity and specificity of results and the inclusion of key studies to ensure comprehensiveness and robustness. The full search strategy of all databases is provided in Multimedia Appendix 1. The final search results were imported into EndNote (version 20, Clarivate) for deduplication and subsequently uploaded into Covidence (Veritas Health Innovation) for collaborative screening.

### Selection of Sources of Evidence

The titles and abstracts of all identified publications were independently evaluated by the reviewers. Article screening was performed using Covidence. After title and abstract screening, the reviewers conducted a full-text screening. Articles that did not include all 3 eligibility criteria were excluded from the review. Each article was evaluated by 2 independent reviewers, and conflicts were resolved by a third independent reviewer.

### Data Charting Process and Data Items

The articles were assessed using a jointly developed form. The data charting was done independently and included the citation metadata (eg, including title and publication year), article type, journal type, study type, year of study, study location, setting, aim, rationale for implementing proposed solutions, technological solutions proposed, care groups involved, handovers studied, current implementation level, and identified advantages and disadvantages.

### Synthesis of Results

Our scoping review synthesizes findings on clinical handover redesigns, emphasizing the use of graphical representations for clarity. This includes an article flowchart following the PRISMA (Preferred Reporting Items for Systematic Reviews and Meta-Analyses) guidelines. The review covers motivations, outcomes, and technological solutions in handover processes, detailed through various charts. The interventions are described in terms of their pros and cons. In terms of technical maturity, technological solutions were categorized as (1) proposing a model or solution, (2) pilot implementation, or (3) full integration into clinical workflows. Technological solutions were grouped by type, with the groups inductively coded based on the extracted data. The review concludes with a maturity graph that highlights temporal trends in the level of maturity of the included studies. A summary of the observations is provided, linking them to the goals of the review and providing insights into the described effectiveness and challenges of the redesigns.

## Results

### Overview

**Figure 1.**
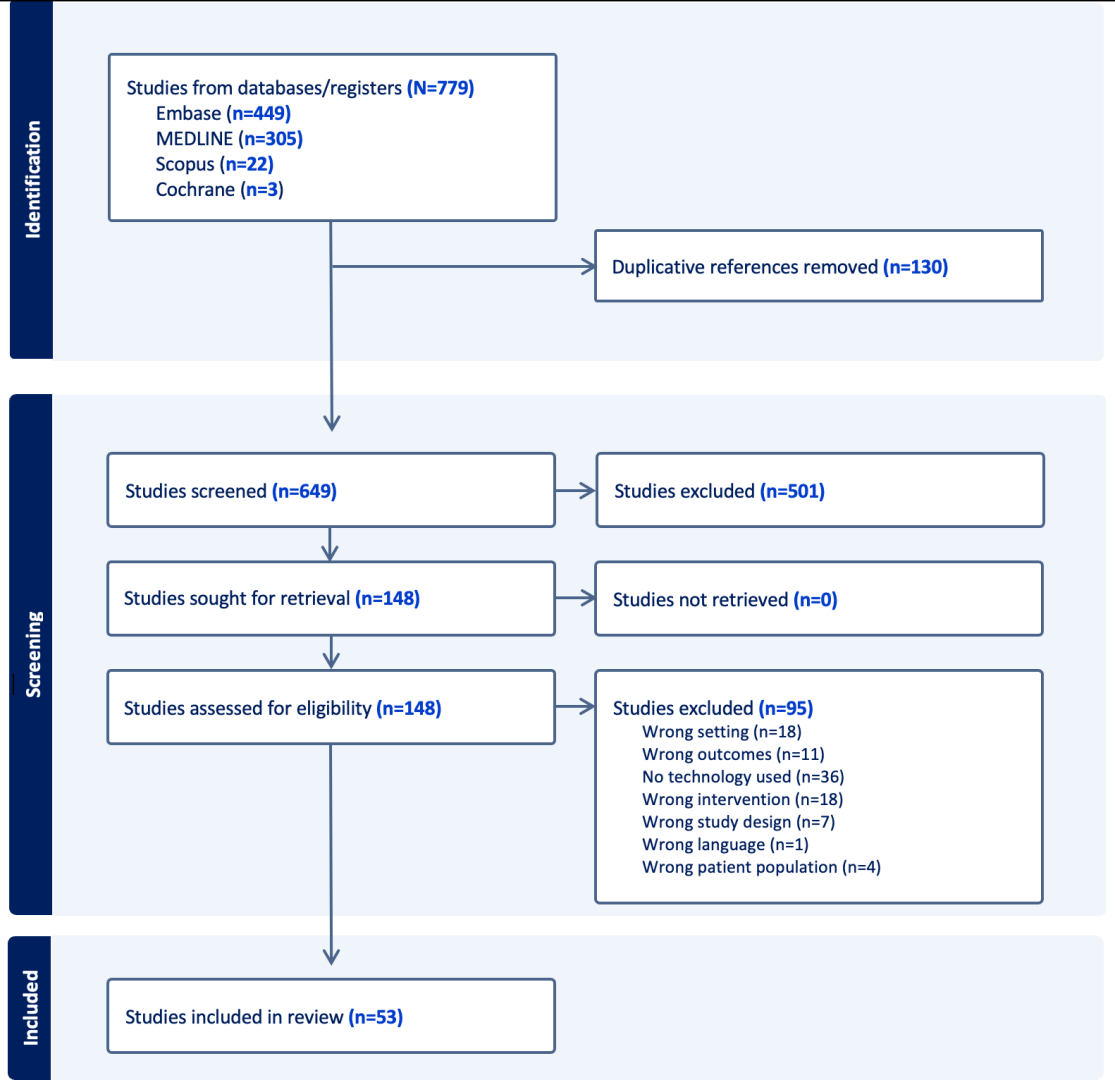
PRISMA (Preferred Reporting Items for Systematic Reviews and Meta-Analyses) flowchart summarizing the article identification, screening, and inclusion process.

A total of 779 studies were retrieved from the databases, of which 130 were automatically removed as duplicates. Of the remaining 649 articles, 501 were excluded during title and abstract screening as they were not within the scope of the study. Of the remaining studies assessed for eligibility (n=148), the majority were excluded since they did not use any technological solution (n=36). A total of 53 articles met the inclusion and exclusion criteria and were used for data extraction (Figure 1 and Multimedia Appendix 2). The rationales behind the included studies are overall quality improvement of the handover process, encompassing multifaceted aspects such as standardization, efficacy enhancement, and error reduction. The settings for these investigations are predominantly perioperative, including operating rooms and postanesthesia care units, with a lesser extent involving intensive care units. The primary interactions occur between physicians, with occasional involvement of nurses and other health care professionals, although the latter are seldom described. A multitude of solutions has been proposed, with the majority exhibiting a relatively low level of technological complexity. Most of these studies are in the pilot phase, with only a few advancing to the stage of full implementation.

### Rationale for Using Technology to Optimize Handover

Several potential justifications have been put forward for the deployment of technology to enhance handover procedures. Among these is the argument that the standardization of communication and information relay will result in improved handover quality [19], greater efficiency [20], greater satisfaction among providers [21], reduced communication and handover errors [22], better outcomes and satisfaction for patients [23], and a clear delineation of responsibility, roles, and authority within the team [24]. These factors are expected to have a positive impact on other areas, such as reducing the length of hospitalization [25] or enhancing trust within the team [24] (Table 2 and Multimedia Appendix 3). Several publications report more than one rationale for improving handover workflows. However, some of the publications do not include a unifying reason for improving handover workflows.

**Table 2.** Proposed rationale for using technology to optimize handover workflows. Some articles stated more than one proposed rationale (N=80).

Proposed rationale	Values, n (%)
Standardize information relay and communication	19 (24)
Reduce communication and handover errors	17 (21)
Increase efficiency of handover process	16 (20)
Improve patient outcomes and satisfaction	12 (15)
Increase provider satisfaction	8 (10)
Others	5 (6)
Delineate responsibility and leadership	3 (4)

### Setting of Handovers

**Figure 2.**
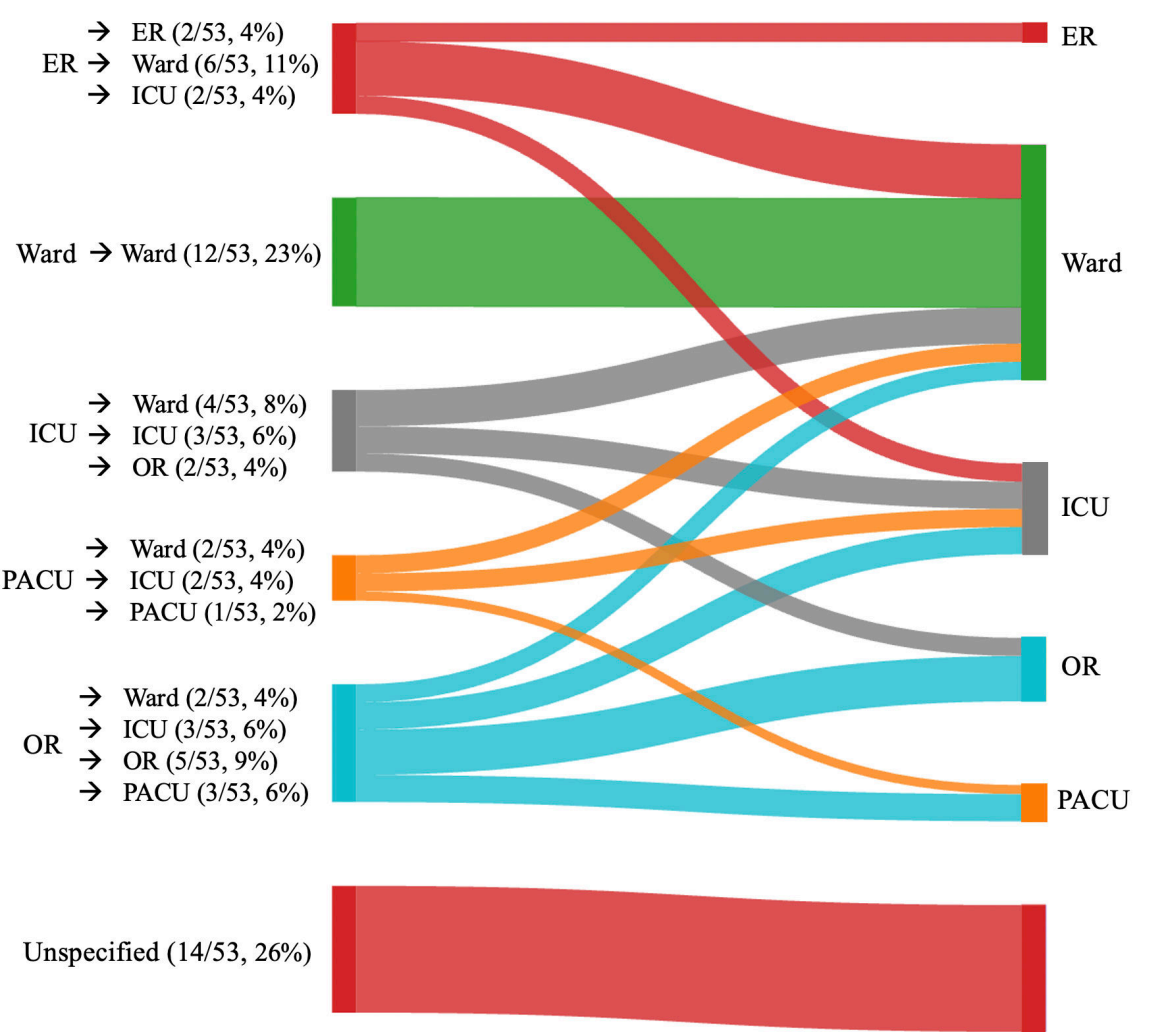
Schematic overview of the hospital departments involved in the clinical handover studies. The total number of settings exceeds the number of included articles, as some studies involved multiple hospital departments. ER: emergency room; ICU: intensive care unit; OR: operating room; PACU: postanesthesia care unit

A large proportion of studies (14/53, 26%) did not specify the exact departments involved in the handovers [20,22,23,26-36]. Among the specified settings, ward-to-ward transitions were the most frequently studied, accounting for 23% (12/53) [37-48]. Emergency room-to-ward handovers followed at 11% (6/53) [24,48-52], while operation-room to operation-room handovers were studied in 9% (5/53) [19,53-56]. The full list of involved hospital departments is available in Figure 2. Of note, the total number of settings exceeds the number of included articles, as some studies involved multiple hospital departments and handover types.

### Care Groups Involved in Handover

The most commonly studied setting was interphysician handovers, which were cited 21 times and accounted for 40% of the total studies (N=53; Table 3) [19,20,22-24,26,27,34,35,38,40,42,43,46,52,57-62]. Of the 53 studies, 10 (19%) focused solely on handovers between nurses [25,29,37,41,45,47,51,63-65]. Handovers exclusively involving both nurses and physicians were examined in 5 (9%) studies [28,30,44,54,66]. Seven (13%) studies explored multidisciplinary handovers involving three or more groups: (1) nurses; (2) physicians; (3) allied health professionals (respiratory therapists, physical and occupational therapists, care coordinators, unit secretaries, dietitians, and pharmacists); and (4) advanced practice providers such as certified registered nurse anesthetists, physician assistants, and nurse practitioners [21,33,39,49,53,56,67]. The full list of care groups involved is available in Table 3.

**Table 3.** Number of studies (n) and proportion (%) of care groups involved for each study (N=53). Studies examining interphysician and internurses’ handovers accounted for approximately two-thirds of study settings.

Involved care group	Values, n (%)
Physicians only	21 (40)
Nurses only	10 (19)
Multidisciplinary (≥3 groups)	7 (13)
Nurses and physicians	5 (9)
Nurses and APPs^a^	3 (6)
Unspecified	3 (6)
Physicians and APPs	2 (4)
Physicians and pharmacists	1 (2)
Physicians and patients	1 (2)

a APPs: advanced practice providers.

### Types of Technological Solutions Deployed

The most prevalent solution, referenced in 15 (28%) of the 53 articles, is a straightforward electronic medical record (EMR)–based checklist (Table 4) [19,20,22-24,26,27,33,41,43,45,53,58,61,62], which refers to an electronic handover template integrated within the hospital’s clinical system, guiding structured documentation of patient information [23]. This is closely followed by electronic versions of existing handover tools, such as I-PASS and (I)SBAR, which are described in 13 (25%) articles [29,34,36,39,40,46,47,51,54,57,59,66,68]. One example is the electronic I-PASS bundle, which digitizes the I-PASS mnemonic to standardize patient handovers, enhance communication clarity, and reduce medical errors through structured, consistent information transfer. Nine (17%) articles describe the use of separate app-based handover tools [28,30,32,38,42,44,56,64,69], for example, a mobile app (Hark) that enables structured, traceable handovers by letting clinicians assign patient-specific tasks with integrated clinical data, real-time notifications, and a full audit trail [44]. Six (11%) articles mention asynchronous handover tools [48,49,52,60,63,70], such as text- or video-based options—for example, a text-based structured electronic handover platform that supports asynchronous patient transfers, allowing teams to review templated clinical information without real-time interaction [52]. Auto-populating handover tools are discussed in 5 (9%) articles [21,25,35,67,71] , which, for instance, include a computerized handover tool linked to auto-populating handover notes from the EMR [34]. Furthermore, 3 (6%) articles [37,55,65] discussed the integration of multiple solutions at once. For example, one article detailed a curriculum that incorporated an adapted I-PASS training session for the neonatal intensive care unit, neonatal simulation scenarios, and a newly developed electronic handover tool [57]. Moreover, 2 (4%) articles assessed the integration of technology for handover in general, thereby not focusing on a specific technological solution but discussing the current technological status quo [31,50].

**Table 4.** Summary of the types of technological solutions studied in the included articles (N=53). Most articles studied simple EMR-based^a^ checklists or electronic versions of existing handover tools.

Types of technological solutions	Values, n (%)
Simple EMR-based checklist	15 (28)
Electronic version of existing tool (I-PASS^b^, (I)SBAR^c^, etc)	13 (25)
Separate app-based handover tool	9 (17)
Asynchronous handover tool (text or video based)	6 (11)
Auto-populating handover tool	5 (9)
Integration of multiple solutions	3 (6)
Unspecified	2 (4)

a EMR: electronic medical record.

b I-PASS: Illness severity, Patient summary, Action items, Situation awareness and contingency planning, Synthesis by receiver.

c (I)SBAR: Introduction, Situation, Background, Assessment, Recommendation.

### Advantages and Disadvantages of Handover Workflow Strategies

The technological solutions investigated in the included studies suggested improvements in the completeness, accuracy, and consistency of critical information transfer, ensuring patient safety, and reducing adverse events attributable to handover errors as potential advantages (Table 5 and Multimedia Appendix 4). The described benefits of these solutions were the enhancement of the quality of communication and handover, streamlining of the handoff preparation, boosting of user satisfaction, shortening of handover length, facilitation of communication with patients’ families and other health care workers, and increasing of the awareness of patients’ conditions.

**Table 5.** Frequency of proposed advantages of technological solutions to improve clinical handovers.

Reported advantages	Values, n
Improved completeness, accuracy, and consistency of critical information during transfer	20
Decreased adverse events due to handover error	8
Improved quality of communication and handover	7
Improved handover preparation efficiency	6
Improved user satisfaction	4
Reduced handover length	3
More likely to communicate with patients’ family and other health care workers	2
Greater awareness of patients’ condition	2
Users felt more prepared for handover	1
Avoided confidentiality breaches with paper printouts	1
Increased standardized handover tool utilization	1
Less time required to understand the patients’ condition	1
Cost-effective solution for ensuring patient safety	1
More convenient handover reporting	1

However, alongside these benefits, certain limitations were repetitively identified (Table 6 and Multimedia Appendix 4). These included challenges related to the generalizability and scalability of the solutions due to the limited scope of analysis, difficulties in ensuring consistent adoption, the necessity for regular updating and user training, limited accessibility and infrastructure, programming limitations hindering the inclusion of necessary elements, restricted opportunities for clarification, the potential reproduction of errors due to prepopulating handover sheets, and the lack of statistically significant improvements in patient outcomes.

**Table 6.** Frequency of proposed disadvantages of technological solutions to improve clinical handovers.

Reported disadvantages	Values, n
Limited generalizability and scalability due to the limited scope of the analysis (eg, specialty-specific, provider-specific, and small dataset)	11
Difficult to ensure consistent adoption	6
Requires regular updating of information, which may take time and increase workload	5
Requires user training, which may pose a steep learning curve for users	5
Limited accessibility for the handover tool due to limited infrastructure	5
Limitations in programming prevent the inclusion of necessary elements	3
Limited opportunities for clarification and requests for additional information	3
Prepopulation of the handover sheet means errors can be reproduced	3
Not completely automated and had to be manually inputted by staff	2
No statistically significant improvement in patient outcomes with these tools	2
Increase in the average duration of verbal handovers	2
Issues with compliance with data privacy on personal devices	1

### Level of Technical Maturity

**Figure 3.**
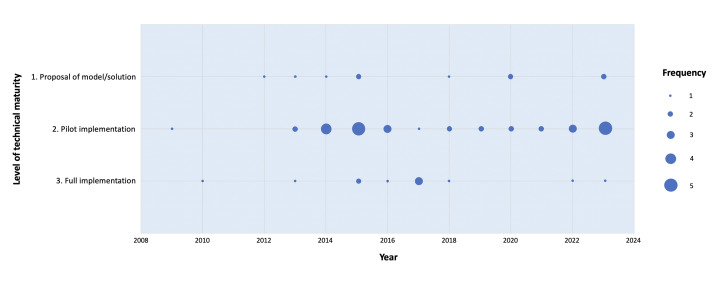
Number of studies published according to their level of maturity and year of publication. No clear temporal trend toward full implementation was found.

For the level of maturity of the technological solutions proposed, out of the 53 studies included, 9 (17%) were proposals of a model or a solution [28-31,37,48,50,53,56], and 11 (21%) were in workflow integration [9,20,22,25,43,51,59,61,62,69,70], while the remaining 33 (62%) articles reported on pilot implementation (Figure 3). No clear time trend toward full implementation was found among the studies.

## Discussion

### Principal Findings

The initial search yielded more than 750 publications reflecting the broad scientific interest in the improvement of clinical handover. After screening and full-text review, this scoping review analyzed 53 articles focusing on technological solutions for improving the clinical handover of inpatients. The review revealed a predominance of low-complexity technologies, such as electronic checklists, with few instances of high-complexity solutions such as natural language processing (NLP). While most studies were in the pilot stage (33/53, 62%), several included documented implementations (11/53, 21%), indicating potential advantages in workflow redesign through technology (Tables 5 and 6). The studied manuscripts described improvements in the completeness, accuracy, and consistency of critical information during patient transfer (n=20), which were identified as the primary advantages of these technological solutions for handover redesign. Additionally, patient safety enhancements through reduced adverse events due to handover errors (n=8) and improved communication quality (n=7) were noted. Other reported benefits included increased handover efficiency (n=6) and enhanced staff satisfaction (n=4) (Multimedia Appendix 4). The latter benefits were documented in only a fraction of the included articles, suggesting the need for further research and broader implementation of advanced technological solutions, mirrored by the low number of studies documenting sustainable implementation. Important considerations include the described disadvantages arising from the integration of technological solutions, including limitations in generalizability and scalability (n=11), inconsistency in adoption (n=6), and inaccessibility of the handover tool (n=5). These challenges may stem from a lack of standardized implementation protocols, varying levels of technological literacy among health care providers, and infrastructural disparities across institutions.

### Advanced Technological Solutions for Handover Redesign

Recent advancements in NLP are not yet well represented in the literature on clinical handovers, which may be due to its novelty. In one included publication using NLP [67], models were shown to perform better at identifying communication content than underlying team communication behaviors. This finding hints at the potential for these models to enhance handover quality and reduce communication errors, ultimately improving patient safety. The study emphasizes the need for further development of these models using larger, real-world datasets to ensure their effectiveness in clinical settings. Additionally, the findings suggest that NLP could be integrated into health care systems to support real-time monitoring and documentation during handovers, ultimately reducing the cognitive load on clinicians and improving care transitions.

Once LLMs become more commonly used in clinical pilot studies and practice, we anticipate their application in handover use cases as well, though major legal and technical barriers exist [72]. For instance, liability in case of malfunctioning resulting in unintended patient harm needs to be clarified. In addition, if classified as a medical device, the European Union Medical Device Regulation [73] requires rigorous validation, risk assessment, and CE marking before routine use. In the United States, the HIPAA (Health Insurance Portability and Accountability Act) requires strict protection of protected health information [74], while the use of an LLM for handovers risks data leakage or improper storage of patient data. Other reasons for the stagnant landscape of handover redesign could be the unintended consequences of more complex solutions, such as undetected confabulation or unchecked LLM implementations, though mitigation strategies are currently under development [75].

Overall, the suitability of an LLM-based approach may vary depending on the clinical context. Automating handovers between different types of health care providers, such as nurses and physicians, can be particularly challenging due to the need to convey distinct types of information [21,28]. In contrast, automation may be more feasible within the same provider group, where shared training and standardized communication practices create a more uniform framework [19,24]. Likewise, handovers between similar units may be easier to implement, as these units follow common operational procedures and have a shared clinical focus [57,59].

The most frequently mentioned challenge for technological solutions was the limited generalizability and scalability of solutions due to a narrow scope of the analysis, for instance, by being specialty-specific [55], provider-specific [41], or only tested using a small sample [19]. LLMs may help to overcome this, as their global training provides them with better generalizability than custom-made solutions tailored toward a specific setting [76].

Meanwhile, attention should be paid to the accuracy and potential biases that may be encoded in the data, so as not to perpetuate health care disparities [77]. While NLP models demonstrate potential in markedly improving handover through specific tasks like identifying communication content, the broader integration of sophisticated technologies using LLMs and AI methods is anticipated to have a more profound impact [78]. However, several studies so far have not shown AI integration to improve diagnostic accuracy or speed up clinical workflows [79]. For AI to be fully leveraged, it must be integrated within the context of redesigned clinical workflows, a task that has yet to be adequately addressed. Before the development and integration of AI solutions, it is vital to determine whether individuals or entities are actively engaged in redesigning workflows for AI to be integrated effectively [80]. Additionally, it is essential to evaluate if such redesigns will genuinely enhance the efficiency of the quality of care. Therefore, the investment in AI should be matched to the investment in clinical workflow redesign to fully realize the potential benefits of these technologies. Without such investment, implementation projects are unlikely to succeed. Continuing workflow errors, particularly those occurring during handovers between health care workers, highlight the urgency of this redesign [19,20,22]. The prevailing approach of adjusting the hammer to the nail must evolve to ensure AI’s success in transforming health care, and handovers in particular.

### Potential Limitations of This Scoping Review

Although an extensive body of literature has been reviewed, the possibility of missing relevant studies cannot be entirely ruled out. To mitigate this, we conducted manual checks and sensitivity analyses, but no additional articles were identified. Furthermore, relevant studies might be inadvertently removed during the screening process, which we aimed to mitigate through independent review by 2 authors. Another limitation is the relatively recent integration of LLMs and AI into this field, meaning that much of the evidence on AI-driven improvements in clinical handovers is likely yet to be published. As a result, this scoping review should be viewed as a snapshot of the current landscape, outlining key challenges that future AI-based research may need to address in their study designs.

### Future Work

This review highlights key gaps in the study of digital handover solutions. One key gap is the variability in detail provided by studies regarding the specific technologies used. Future studies should aim to provide clearer descriptions of the tools being evaluated to facilitate comparisons across different settings. Another critical area for future research is the need for more objective, quantitative measures to assess the impact of these handover tools. Subsequently, a systematic review (with meta-analysis) could assess the effectiveness of interventions and the quality of evidence. Most studies in this review relied on qualitative feedback from health care professionals, which limits comparability and generalizability. Long-term studies are also needed to assess the lasting impact of these tools on communication and workload. Comparative studies across various health care settings and specialties could also help determine how well these solutions scale and adapt to different clinical environments. Further research is also needed to explore the scalability and real-world effectiveness of AI solutions and to address the infrastructural and regulatory challenges that impede their widespread adoption.

Crucially, successful adoption of these technologies may require concurrent redesign of clinical handovers to fully realize their benefits and ensure that they improve both the efficiency and safety of care. The prevailing approach of simply adapting technology to fit existing workflows must evolve if AI is to truly transform health care and improve patient outcomes.

### Conclusion

This scoping review underscores the substantial interest in improving clinical handovers through technological solutions, particularly low-complexity technologies such as electronic checklists. While the literature describes solutions that promise to improve the accuracy, completeness, and communication of information during patient handovers, wider implementation and sustainability remain limited. Key challenges included limited generalizability, scalability, and accessibility of solutions, along with difficulties in adoption, maintenance, and training. While advanced technologies such as NLP and AI may have the potential to enhance handover processes, no studies were identified that report their integration into clinical practice.

## Supplementary material

10.2196/70358 Multimedia Appendix 1 Search strategy for article identification from the 4 queried databases.

10.2196/70358 Multimedia Appendix 2 Bibliographic list of included articles.

10.2196/70358 Multimedia Appendix 3 Summary of extracted articles.

10.2196/70358 Multimedia Appendix 4 List of common advantages and disadvantages afforded by technological solutions.

10.2196/70358 Checklist 1 PRISMA-ScR (Preferred Reporting Items for Systematic Reviews and Meta-Analyses extension for Scoping Reviews) checklist.

## Abbreviations

(I)SBAR: Introduction, Situation, Background, Assessment, Recommendation
AI: artificial intelligence
EMR: electronic medical record
HIPAA: Health Insurance Portability and Accountability Act
I-PASS: Illness severity, Patient summary, Action items, Situation awareness and contingency planning, Synthesis by receiver
JBI: Joanna Briggs Institute
LLM: large language model
MeSH: Medical Subject Headings
ML: machine learning
NLP: natural language processing
PCC: Population, Concept, and Context framework
PRISMA: Preferred Reporting Items for Systematic Reviews and Meta-Analyses
PRISMA-ScR: Preferred Reporting Items for Systematic Reviews and Meta-Analyses extension for Scoping Reviews

## References

[1] Yu F, Moehring A, Banerjee O, Salz T, Agarwal N, Rajpurkar P (2024). Heterogeneity and predictors of the effects of AI assistance on radiologists. Nat Med.

[2] Desmedt M, Ulenaers D, Grosemans J, Hellings J, Bergs J (2021). Clinical handover and handoff in healthcare: a systematic review of systematic reviews. Int J Qual Health Care.

[3] Zayas-Cabán T, Okubo TH, Posnack S (2022). Priorities to accelerate workflow automation in health care. J Am Med Inform Assoc.

[4] National Academies of Sciences, Engineering, and Medicine, National Academy of Medicine, Committee on Systems Approaches to Improve Patient Care by Supporting Clinician Well-Being (2019). Taking Action Against Clinician Burnout: A Systems Approach to Professional Well-Being.

[5] Bywaters E, Calvert S, Eccles S (2020). Safe handover: safe patients. University of Salford Repository.

[6] Cohen MD, Hilligoss PB (2010). The published literature on handoffs in hospitals: deficiencies identified in an extensive review. Qual Saf Health Care.

[7] Raeisi A, Rarani MA, Soltani F (2019). Challenges of patient handover process in healthcare services: a systematic review. J Educ Health Promot.

[8] Foster S, Manser T (2012). The effects of patient handoff characteristics on subsequent care: a systematic review and areas for future research. Acad Med.

[9] Starmer AJ, O’Toole JK, Rosenbluth G (2014). Development, implementation, and dissemination of the I-PASS handoff curriculum: a multisite educational intervention to improve patient handoffs. Acad Med.

[10] Astier A, Carlet J, Hoppe-Tichy T (2020). What is the role of technology in improving patient safety? A French, German and UK healthcare professional perspective. J Patient Saf Risk Manag.

[11] Reddy V, Brumpton L (2021). Digital-driven service improvement during the COVID-19 pandemic. Paediatr Child Health (Oxford).

[12] Solari-Twadell PA, Flinter M, Rambur B (2022). The impact of the COVID-19 pandemic on the future of telehealth in primary care. Nurs Outlook.

[13] Ozkaynak M, Unertl K, Johnson S, Brixey J, Haque SN, Finnell JT, Dixon BE (2022). Clinical Informatics Study Guide: Text and Review.

[14] Singh G, Singh R, Singh A, Raj D (2013). Improvement of workflow and processes to ease and enrich meaningful use of health information technology. Adv Med Educ Pract.

[15] Tricco AC, Lillie E, Zarin W (2018). PRISMA Extension for Scoping Reviews (PRISMA-ScR): checklist and explanation. Ann Intern Med.

[16] Peters MDJ, Marnie C, Tricco AC (2021). Updated methodological guidance for the conduct of scoping reviews. JBI Evid Implement.

[17] Improved clinical workflows through technological solutions – fact or fiction? A scoping review of inpatient handovers. Open Science Framework.

[18] Pollock D, Peters MDJ, Khalil H (2023). Recommendations for the extraction, analysis, and presentation of results in scoping reviews. JBI Evid Synth.

[19] Agarwala AV, Firth PG, Albrecht MA, Warren L, Musch G (2015). An electronic checklist improves transfer and retention of critical information at intraoperative handoff of care. Anesth Analg.

[20] Anderson J, Shroff D, Curtis A (2010). The Veterans Affairs shift change physician-to-physician handoff project. Jt Comm J Qual Patient Saf.

[21] Bell E, Benefield D, Vollenweider A, Wilson K, Warren LL, Aroke EN (2023). Improving communication between ICU nurses and anesthesia providers using a standardized handoff protocol. J Perianesth Nurs.

[22] Cheng DR, Liddle J, Mailes E, South M (2017). Impact of an integrated electronic handover tool on pediatric junior medical staff (JMS) handover. Int J Med Inform.

[23] Graham KL, Marcantonio ER, Huang GC, Yang J, Davis RB, Smith CC (2013). Effect of a systems intervention on the quality and safety of patient handoffs in an internal medicine residency program. J Gen Intern Med.

[24] Smith CJ, Buzalko RJ, Anderson N (2018). Evaluation of a novel handoff communication strategy for patients admitted from the emergency department. West J Emerg Med.

[25] Sexton P, Whiteman K, George EL, Fanning M, Stephens K (2022). Improving PACU throughput using an electronic dashboard: a quality improvement initiative. J Perianesth Nurs.

[26] Atkinson CT, Mir HR (2015). Development of an orthopaedic handover system to improve communication for inpatient care. Curr Orthop Pract.

[27] Jose Santana M, de Grood C, Eso K (2015). Physicians’ experience adopting the electronic transfer of care communication tool: barriers and opportunities. J Multidiscip Healthc.

[28] Flemming D, Hübner U (2013). How to improve change of shift handovers and collaborative grounding and what role does the electronic patient record system play? Results of a systematic literature review. Int J Med Inform.

[29] Hou YH, Lu LJ, Lee PH, Chang IC (2019). Positive impacts of electronic hand-off systems designs on nurses’ communication effectiveness. J Nurs Manag.

[30] Johnston MJ, King D, Arora S (2014). Requirements of a new communication technology for handover and the escalation of patient care: a multi-stakeholder analysis. J Eval Clin Pract.

[31] Patterson ES (2012). Technology support of the handover: promoting observability, flexibility and efficiency. BMJ Qual Saf.

[32] Santana MJ, Holroyd-Leduc J, Flemons WW (2014). The seamless transfer of care: a pilot study assessing the usability of an electronic transfer of care communication tool. Am J Med Qual.

[33] Schuster KM, Jenq GY, Thung SF (2014). Electronic handoff instruments: a truly multidisciplinary tool?. J Am Med Inform Assoc.

[34] Skaret MM, Weaver TD, Humes RJ, Carbone TV, Grasso IA, Kumar H (2019). Automation of the I-PASS tool to improve transitions of care. J Healthc Qual.

[35] Starmer AJ, Sectish TC, Simon DW (2013). Rates of medical errors and preventable adverse events among hospitalized children following implementation of a resident handoff bundle. JAMA.

[36] Franco Vega MC, Ait Aiss M, Smith M (2023). Improving handoff with the implementation of I-PASS at a tertiary oncology hospital. BMJ Open Qual.

[37] Bukoh MX, Siah CJR (2020). A systematic review on the structured handover interventions between nurses in improving patient safety outcomes. J Nurs Manag.

[38] Clarke CN, Patel SH, Day RW (2017). Implementation of a standardized electronic tool improves compliance, accuracy, and efficiency of trainee-to-trainee patient care handoffs after complex general surgical oncology procedures. Surgery.

[39] Cornell P, Townsend-Gervis M, Vardaman JM, Yates L (2014). Improving situation awareness and patient outcomes through interdisciplinary rounding and structured communication. J Nurs Adm.

[40] García Roig C, Viard MV, García Elorrio E (2020). Implementation of a structured patient handoff between health care providers at a private facility in the Autonomous City of Buenos Aires. Arch Argent Pediatr.

[41] Johnson M, Sanchez P, Zheng C (2016). The impact of an integrated nursing handover system on nurses’ satisfaction and work practices. J Clin Nurs.

[42] Nabors C, Patel D, Khera S (2015). Improving resident morning sign-out by use of daily events reports. J Patient Saf.

[43] Coughlan JJ, Mross T, Gul F (2018). Implementing an electronic clinical handover system in a university teaching hospital. Ir J Med Sci.

[44] Patel B, Johnston M, Cookson N, King D, Arora S, Darzi A (2016). Interprofessional communication of clinicians using a mobile phone app: a randomized crossover trial using simulated patients. J Med Internet Res.

[45] Taylor JS (2015). Improving patient safety and satisfaction with standardized bedside handoff and walking rounds. Clin J Oncol Nurs.

[46] Walia J, Qayumi Z, Khawar N (2016). Physician transition of care: benefits of I-PASS and an electronic handoff system in a community pediatric residency program. Acad Pediatr.

[47] Zhou J, Zhang F, Wang H (2022). Quality and efficiency of a standardized e-handover system for pediatric nursing: a prospective interventional study. J Nurs Manag.

[48] Delardes B, McLeod L, Chakraborty S, Bowles KA (2020). What is the effect of electronic clinical handovers on patient outcomes? A systematic review. Health Informatics J.

[49] Benham-Hutchins MM, Effken JA (2010). Multi-professional patterns and methods of communication during patient handoffs. Int J Med Inform.

[50] Popovici I, Morita PP, Doran D (2015). Technological aspects of hospital communication challenges: an observational study. Int J Qual Health Care.

[51] Potts L, Ryan C, Diegel-Vacek L, Murchek A (2018). Improving patient flow from the emergency department utilizing a standardized electronic nursing handoff process. J Nurs Adm.

[52] Sanchez LD, Chiu DT, Nathanson L (2017). A model for electronic handoff between the emergency department and inpatient units. J Emerg Med.

[53] Abraham J, Duffy C, Kandasamy M, France D, Greilich P (2023). An evidence synthesis on perioperative handoffs: a call for balanced sociotechnical solutions. Int J Med Inform.

[54] Hong Mershon B, Vannucci A, Bryson T (2021). A collaborative partnership between the multicenter handoff collaborative and an electronic health record vendor. Appl Clin Inform.

[55] Lai YH, Gui JL, Arif A, Okorozo A, Patten D (2023). Design and implementation of a structured application-based intraoperative handoff tool for anesthesia care teams: a quality improvement approach. Middle East J Anesthiol.

[56] Sparling JL, Hong Mershon B, Abraham J (2023). Perioperative handoff enhancement opportunities through technology and artificial intelligence: a narrative review. Jt Comm J Qual Patient Saf.

[57] Quinones Cardona V, LaBadie A, Cooperberg DB, Zubrow A, Touch SM (2021). Improving the neonatal team handoff process in a level IV NICU: reducing interruptions and handoff duration. BMJ Open Qual.

[58] Dubosh NM, Carney D, Fisher J, Tibbles CD (2014). Implementation of an emergency department sign-out checklist improves transfer of information at shift change. J Emerg Med.

[59] Panesar RS, Albert B, Messina C, Parker M (2016). The effect of an electronic SBAR communication tool on documentation of acute events in the pediatric intensive care unit. Am J Med Qual.

[60] Westaway S, Webber T, Gluck S, Sundararajan K (2022). Lost in relocation: longitudinal surveys evaluating the effectiveness of ICU to ward handover after the introduction of an electronic patient record. Hosp Pract.

[61] Yee KC, Wong MC, Turner P (2013). Understanding how clinical judgement and communicative practices interact with the use of an electronic clinical handover system. Stud Health Technol Inform.

[62] Yanni E, Calaman S, Wiener E, Fine JS, Sagalowsky ST (2023). Implementation of ED I-PASS as a standardized handoff tool in the pediatric emergency department. J Healthc Qual.

[63] Lieng MK, Siefkes HM, Rosenthal JL (2019). Telemedicine for interfacility nurse handoffs. Pediatr Crit Care Med.

[64] Schmidt T, Kocher DR, Mahendran P, Denecke K (2019). Dynamic pocket card for implementing ISBAR in shift handover communication. Stud Health Technol Inform.

[65] O’Neill K, Powell M, Lovell T (2023). Improving the handover of complex trauma patients by implementing a standardised process. Aust Crit Care.

[66] Shah AC, Herstein AR, Flynn-O’Brien KT, Oh DC, Xue AH, Flanagan MR (2019). Six Sigma methodology and postoperative information reporting: a multidisciplinary quality improvement study with interrupted time-series regression. J Surg Educ.

[67] Mayes E, Gehlbach JA, Jeziorczak PM, Wooldridge AR (2023). Machine learning to operationalize team cognition: a case study of patient handoffs. Hum Factors Healthc.

[68] Benton SE, Hueckel RM, Taicher B, Muckler VC (2020). Usability assessment of an electronic handoff tool to facilitate and improve postoperative communication between anesthesia and intensive care unit staff. Comput Inform Nurs.

[69] Oakley B, Hunter JB (2017). Implementing an electronic patient handover system. Br J Hosp Med.

[70] Weinger MB, Slagle JM, Kuntz AH (2015). A multimodal intervention improves postanesthesia care unit handovers. Anesth Analg.

[71] Improving Communication Between ICU Nurses and Anesthesia Providers Using a Standardized Handoff Protocol. Journal of perianesthesia nursing official journal of the American Society of PeriAnesthesia Nurses 2022;():2022.

[72] Ullah E, Parwani A, Baig MM, Singh R (2024). Challenges and barriers of using large language models (LLM) such as ChatGPT for diagnostic medicine with a focus on digital pathology: a recent scoping review. Diagn Pathol.

[73] Regulation (EU) 2017/745. EUR-Lex.

[74] (2008). Summary of the HIPAA privacy rule. US Department of Health and Human Services.

[75] Farquhar S, Kossen J, Kuhn L, Gal Y (2024). Detecting hallucinations in large language models using semantic entropy. Nature New Biol.

[76] Patil R, Gudivada V (2024). A review of current trends, techniques, and challenges in large language models (LLMs). Appl Sci (Basel).

[77] Celi LA, Cellini J, Charpignon ML (2022). Sources of bias in artificial intelligence that perpetuate healthcare disparities: a global review. PLOS Digit Health.

[78] Coiera E, Kocaballi B, Halamka J, Laranjo L (2018). The digital scribe. NPJ Digit Med.

[79] Menzies SW, Sinz C, Menzies M (2023). Comparison of humans versus mobile phone-powered artificial intelligence for the diagnosis and management of pigmented skin cancer in secondary care: a multicentre, prospective, diagnostic, clinical trial. Lancet Digit Health.

[80] Alowais SA, Alghamdi SS, Alsuhebany N (2023). Revolutionizing healthcare: the role of artificial intelligence in clinical practice. BMC Med Educ.

