# Supplementary material for: Technological Solutions to Improve Inpatient Handover in the Era of Artificial Intelligence: Scoping Review
Source: J Med Internet Res. 2025 Jul 31;27:e70358. doi: 10.2196/70358 (PMC12312997; doi:10.2196/70358)
Supplement: Multimedia Appendix 1 [file jmir-v27-e70358-s001.docx]

**Search strategy for article identification from the 4 queried databases**

**Medline Ovid**

1. exp "Workflow"/
2. exp Patient Handoff/ or exp Patient Transfer/
3. exp Process Assessment, Health Care/
4. ("work flow*" or "workflow*" or process*).ti.
5. ("work flow*" or "workflow*").ab. /freq=2
6. or/1-5 [workflow terms]
7. exp *Quality Improvement/ or exp Process Assessment, Health Care/ or exp Quality Assurance, Health Care/
8. (design* or redesign* or improv* or engineer* or re-engineer*).ti.
9. (design* or redesign* or improv* or engineer* or re-engineer* or "process map*").ab. /freq=2
10. or/7-9 [redesign]
11. exp patient care team/ or exp nursing, team/ or *primary health care/ or exp health personnel/ or exp *Practice Management, Medical/
12. (clinic* or doctor* or physician* or resident* or nurs* or pharmacist*).ti,kf.
13. 11 or 12 [provider terms]
14. 6 and 10 and 13
15. exp *Communication/
16. exp *Patient Handoff/ or exp *Patient Transfer/
17. (communicat* or discuss* or handoff* or "patient transfer*").ab,kf,ti. /freq=3
18. 15 or 16 or 17 [communication terms]
19. 14 and 18

**Embase Ovid**

1. exp *workflow/
2. exp patient transport/ or exp hospital discharge/ or exp patient transfer/
3. exp health care quality/ or exp process monitoring/
4. ("work flow*" or "workflow*" or process*).ti.
5. ("work flow*" or "workflow*").ab. /freq=2
6. or/1-5 [workflow terms]
7. exp *health care quality/ or exp *process monitoring/ or exp *quality control/
8. (design* or redesign* or improv* or engineer* or re-engineer*).ti.
9. (design* or redesign* or improv* or engineer* or re-engineer* or "process map*").ab. /freq=2
10. or/7-9 [redesign]
11. exp *health care personnel/ or exp *medical staff/ or exp *nursing staff/ or exp *primary health care/ or exp *medical practice/
12. (clinic* or doctor* or physician* or resident* or nurs* or pharmacist*).ti,kw.
13. 11 or 12 [provider terms]
14. 6 and 10 and 13
15. exp *interpersonal communication/
16. exp *patient transport/ or exp *hospital discharge/ or exp *patient transfer/
17. (communicat* or discuss* or handoff* or "patient transfer*").ab,kw,ti. /freq=3
18. 15 or 16 or 17 [communication terms]
19. 14 and 18

**Scopus**

(INDEXTERMS("workflow") OR INDEXTERMS("patient handoff") OR INDEXTERMS("patient transfer") OR INDEXTERMS("process assessment") OR TITLE("work flow*" OR "workflow*" OR process*) OR ABS("work flow*" OR "workflow*")) AND (INDEXTERMS("quality improvement") OR INDEXTERMS("process assessment") OR INDEXTERMS("quality assurance") OR TITLE(design* OR redesign* OR improv* OR engineer* OR re-engineer*) OR ABS(design* OR redesign* OR improv* OR engineer* OR re-engineer* OR "process map*")) AND (INDEXTERMS("patient care team") OR INDEXTERMS("nursing team") OR INDEXTERMS("primary health care") OR INDEXTERMS("health personnel") OR INDEXTERMS("practice management") OR TITLE(clinic* OR doctor* OR physician* OR resident* OR nurs* OR pharmacist*) OR KEY(clinic* OR doctor* OR physician* OR resident* OR nurs* OR pharmacist*)) AND (INDEXTERMS("communication") OR INDEXTERMS("patient handoff") OR INDEXTERMS("patient transfer") OR TITLE(communicat* OR discuss* OR handoff* OR "patient transfer*") OR KEY(communicat* OR discuss* OR handoff* OR "patient transfer*") OR ABS(communicat* OR discuss* OR handoff* OR "patient transfer*"))

**Cochrane**

#1 explode all trees [Workflow]

#2 explode all trees [Patient Handoff] OR explode all trees [Patient Transfer]

#3 explode all trees [Process Assessment, Health Care]

#4 ("work flow*" or "workflow*" or process*):ti

#5 ("work flow*" or "workflow*"):ab

#6 #1 OR #2 OR #3 OR #4 OR #5

#7 explode all trees [Quality Improvement] OR explode all trees [Process Assessment, Health Care] OR explode all trees [Quality Assurance, Health Care]

#8 (design* or redesign* or improv* or engineer* or re-engineer*):ti

#9 (design* or redesign* or improv* or engineer* or re-engineer* or "process map*"):ab

#10 #7 OR #8 OR #9

#11 explode all trees [Patient Care Team] OR explode all trees [Nursing, Team] OR [Primary Health Care] OR explode all trees [Health Personnel] OR [Practice Management, Medical]

#12 (clinic* or doctor* or physician* or resident* or nurs* or pharmacist*):ti,kw

#13 #11 OR #12

#14 #6 AND #10 AND #13

#15 [Communication]

#16 [Patient Handoff] OR [Patient Transfer]

#17 (communicat* or discuss* or handoff* or "patient transfer*"):ti,ab,kw

#18 #15 OR #16 OR #17

#19 #14 AND #18
