# Supplementary material for: Technological Solutions to Improve Inpatient Handover in the Era of Artificial Intelligence: Scoping Review
Source: J Med Internet Res. 2025 Jul 31;27:e70358. doi: 10.2196/70358 (PMC12312997; doi:10.2196/70358)
Supplement: Multimedia Appendix 2 [file jmir-v27-e70358-s002.docx]

**Bibliographic list of included articles**

| **#** | **Title** | **Authors** | **Year** | **Journal** |
| --- | --- | --- | --- | --- |
| 1 | An electronic checklist improves transfer and retention of critical information at intraoperative handoff of care | Agarwala, A. V.; Firth, P. G.; Albrecht, M. A.; Warren, L.; Musch, G. | 2015 | Anesthesia and Analgesia |
| 2 | The Veterans Affairs shift change physician-to-physician handoff project | Anderson, Jaclyn; Shroff, Divya; Curtis, Ann; Eldridge, Noel; Cannon, Katrina; Karnani, Rajil; Abrams, Thad; Kaboli, Peter | 2010 | Joint Commission journal on quality and patient safety |
| 3 | Interdisciplinary mistrust, communication breakdowns cited in survey of ED handoffs | Anonymous | 2015 | ED management : the monthly update on emergency department management |
| 4 | Development of an orthopaedic handover system to improve communication for inpatient care | Atkinson, C. T.; Mir, H. R. | 2015 | Current Orthopaedic Practice |
| 5 | Improving Communication Between ICU Nurses and Anesthesia Providers Using a Standardized Handoff Protocol | Bell, E.; Benefield, D.; Vollenweider, A.; Wilson, K.; Warren, L. L.; Aroke, E. N. | 2022 | Journal of perianesthesia nursing : official journal of the American Society of PeriAnesthesia Nurses |
| 6 | Multi-professional patterns and methods of communication during patient handoffs | Benham-Hutchins, M. M.; Effken, J. A. | 2010 | International Journal of Medical Informatics |
| 7 | Usability Assessment of an Electronic Handoff Tool to Facilitate and Improve Postoperative Communication Between Anesthesia and Intensive Care Unit Staff | Benton, Samuel Edison; Hueckel, Remi M.; Taicher, Brad; Muckler, Virginia C. | 2020 | Computers, informatics, nursing : CIN |
| 8 | A systematic review on the structured handover interventions between nurses in improving patient safety outcomes | Bukoh, Ming Xuan; Siah, Chiew-Jiat Rosalind | 2020 | Journal of nursing management |
| 9 | Improving the neonatal team handoff process in a level IV NICU: Reducing interruptions and handoff duration | Cardona, V. Q.; Labadie, A.; Cooperberg, D. B.; Zubrow, A.; Touch, S. M. | 2021 | BMJ Open Quality |
| 10 | Impact of an integrated electronic handover tool on pediatric junior medical staff (JMS) handover | Cheng, Daryl R.; Liddle, James; Mailes, Emily; South, Mike | 2017 | International journal of medical informatics |
| 11 | Implementation of a standardized electronic tool improves compliance, accuracy, and efficiency of trainee-to-trainee patient care handoffs after complex general surgical oncology procedures | Clarke, C. N.; Patel, S. H.; Day, R. W.; George, S.; Sweeney, C.; Monetes De Oca, G. A.; Aiss, M. A.; Grubbs, E. G.; Bednarski, B. K.; Lee, J. E.; Bodurka, D. C.; Skibber, J. M.; Aloia, T. A. | 2017 | Surgery (United States) |
| 12 | Improving situation awareness and patient outcomes through interdisciplinary rounding and structured communication | Cornell, Paul; Townsend-Gervis, Mary; Vardaman, James M.; Yates, Lauren | 2014 | The Journal of nursing administration |
| 13 | Implementing an electronic clinical handover system in a university teaching hospital | Coughlan, J. J.; Mross, T.; Gul, F.; Abbott, A.; Say, R.; Nawaz, A.; O'Brien, C.; Liston, R. | 2018 | Irish Journal of Medical Science |
| 14 | Physicians' experience adopting the electronic transfer of care communication tool: Barriers and opportunities | de Grood, C.; Eso, K.; Santana, M. J. | 2015 | Journal of Multidisciplinary Healthcare |
| 15 | What is the effect of electronic clinical handovers on patient outcomes? A systematic review | Delardes, Belinda; McLeod, Lisa; Chakraborty, Samantha; Bowles, Kelly-Ann | 2020 | Health informatics journal |
| 16 | Implementation of an emergency department sign-out checklist improves transfer of information at shift change | Dubosh, Nicole M.; Carney, Dylan; Fisher, Jonathan; Tibbles, Carrie D. | 2014 | The Journal of emergency medicine |
| 17 | How to improve change of shift handovers and collaborative grounding and what role does the electronic patient record system play? Results of a systematic literature review | Flemming, Daniel; Hubner, Ursula | 2013 | International journal of medical informatics |
| 18 | Implementation of a structured patient handoff between health care providers at a private facility in the Autonomous City of Buenos Aires | Garcia Roig, Cristian; Viard, Maria V.; Garcia Elorrio, Ezequiel; Suarez Anzorena, Ines; Jorro Baron, Facundo; Colaboradoras | 2020 | Implementacion de un pase estructurado de pacientes entre profesionales en una institucion privada de la Ciudad Autonoma de Buenos Aires. |
| 19 | Effect of a systems intervention on the quality and safety of patient handoffs in an internal medicine residency program | Graham, K. L.; Marcantonio, E. R.; Huang, G. C.; Yang, J.; Davis, R. B.; Smith, C. C. | 2013 | Journal of General Internal Medicine |
| 20 | A Collaborative Partnership between the Multicenter Handoff Collaborative and an Electronic Health Record Vendor | Hong Mershon, B.; Vannucci, A.; Bryson, T.; Lin, F.; Greilich, P. E.; Dear, G.; Guffey, P.; Agarwala, A. | 2021 | Applied clinical informatics |
| 21 | Positive Impacts of Electronic hand-off systems designs on Nurses' communication effectiveness | Hou, Ying-Hui; Lu, Li-Jung; Lee, Pei-Hsuan; Chang, I. Chiu | 2019 | Journal of nursing management |
| 22 | The impact of an integrated nursing handover system on nurses' satisfaction and work practices | Johnson, Maree; Sanchez, Paula; Zheng, Catherine | 2016 | Journal of clinical nursing |
| 23 | Requirements of a new communication technology for handover and the escalation of patient care: A multi-stakeholder analysis | Johnston, M. J.; King, D.; Arora, S.; Cooper, K.; Panda, N. A.; Gosling, R.; Singh, K.; Sanders, B.; Cox, B.; Darzi, A. | 2014 | Journal of Evaluation in Clinical Practice |
| 24 | Telemedicine for Interfacility Nurse Handoffs | Lieng, Monica K.; Siefkes, Heather M.; Rosenthal, Jennifer L.; Sauers-Ford, Hadley S.; Mouzoon, Jamie L.; Sigal, Ilana S.; Dayal, Parul; Chen, Shelby T.; McBeth, Cheryl L.; Dial, Sandie; Dizon, Genevieve; Dannewitz, Haley E.; Kozycz, Kiersten; Jennings-Hill, Torryn L.; Martinson, Jennifer M.; Huerta, Julia K.; Pons, Emily A.; Vance, Nicole; Warnock, Breanna N.; Marcin, James P. | 2019 | Pediatric critical care medicine : a journal of the Society of Critical Care Medicine and the World Federation of Pediatric Intensive and Critical Care Societies |
| 25 | Improving resident morning sign-out by use of daily events reports | Nabors, Christopher; Patel, Dhruv; Khera, Sahil; Kolte, Dhaval; Gupta, Ridhi; Balasubramaniyam, Nivas; Ambrale, Samir; Mukhi, Nikhil; Lamba, Rajat; Ramachandraiah, Vidya; Subramanian, Kathir; Syed, Rashid; Nam, Kyung Hun; Dardi, Inderpreet Kaur; Bommena, Shoma; Mittal, Varun; Peterson, Stephen J. | 2015 | Journal of patient safety |
| 26 | Implementing an electronic patient handover system | Oakley, Ben; Hunter, James B. | 2017 | British journal of hospital medicine (London, England : 2005) |
| 27 | The Effect of an Electronic SBAR Communication Tool on Documentation of Acute Events in the Pediatric Intensive Care Unit | Panesar, Rahul S.; Albert, Ben; Messina, Catherine; Parker, Margaret | 2016 | American journal of medical quality : the official journal of the American College of Medical Quality |
| 28 | Interprofessional Communication of Clinicians Using a Mobile Phone App: A Randomized Crossover Trial Using Simulated Patients | Patel, Bhavesh; Johnston, Maximilian; Cookson, Natalie; King, Dominic; Arora, Sonal; Darzi, Ara | 2016 | Journal of medical Internet research |
| 29 | Technology support of the handover: Promoting observability, flexibility and efficiency | Patterson, E. S. | 2012 | BMJ Quality and Safety |
| 30 | Technological aspects of hospital communication challenges: an observational study | Popovici, Ilinca; Morita, Plinio P.; Doran, Diane; Lapinsky, Stephen; Morra, Dante; Shier, Ashleigh; Wu, Robert; Cafazzo, Joseph A. | 2015 | International journal for quality in health care : journal of the International Society for Quality in Health Care |
| 31 | Improving Patient Flow From the Emergency Department Utilizing a Standardized Electronic Nursing Handoff Process | Potts, Lisa; Ryan, Catherine; Diegel-Vacek, Lauren; Murchek, Alisa | 2018 | The Journal of nursing administration |
| 32 | A Model for Electronic Handoff Between the Emergency Department and Inpatient Units | Sanchez, L. D.; Chiu, D. T.; Nathanson, L.; Horng, S.; Wolfe, R. E.; Zeidel, M. L.; Boyd, K.; Tibbles, C.; Calder, S.; Dufresne, J.; Yang, J. J. | 2017 | Journal of Emergency Medicine |
| 33 | The seamless transfer of care: a pilot study assessing the usability of an electronic transfer of care communication tool | Santana, M. J.; Holroyd-Leduc, J.; Flemons, W. W.; O'Beirne, M.; White, D.; Clayden, N.; Forster, A. J.; Ghali, W. A. | 2014 | American journal of medical quality : the official journal of the American College of Medical Quality |
| 34 | Dynamic Pocket Card for Implementing ISBAR in Shift Handover Communication | Schmidt, T.; Kocher, D. R.; Mahendran, P.; Denecke, K. | 2019 | Studies in health technology and informatics |
| 35 | Electronic handoff instruments: a truly multidisciplinary tool? | Schuster, Kevin M.; Jenq, Grace Y.; Thung, Stephen F.; Hersh, David C.; Nunes, Judy; Silverman, David G.; Horwitz, Leora I. | 2014 | Journal of the American Medical Informatics Association : JAMIA |
| 36 | Improving PACU Throughput Using an Electronic Dashboard: A Quality Improvement Initiative | Sexton, P.; Whiteman, K.; George, E. L.; Fanning, M.; Stephens, K. | 2022 | Journal of perianesthesia nursing : official journal of the American Society of PeriAnesthesia Nurses |
| 37 | Six Sigma Methodology and Postoperative Information Reporting: A Multidisciplinary Quality Improvement Study With Interrupted Time-Series Regression | Shah, Aalap C.; Herstein, Andrew R.; Flynn-O'Brien, Katherine T.; Oh, Daniel C.; Xue, Anna H.; Flanagan, Meghan R. | 2019 | Journal of surgical education |
| 38 | Automation of the I-PASS Tool to Improve Transitions of Care | Skaret, Michael M.; Weaver, Travis D.; Humes, Ross J.; Carbone, Thomas V.; Grasso, Ian A.; Kumar, Harjinder | 2019 | Journal for healthcare quality : official publication of the National Association for Healthcare Quality |
| 39 | Rates of medical errors and preventable adverse events among hospitalized children following implementation of a resident handoff bundle | Starmer, Amy J.; Sectish, Theodore C.; Simon, Dennis W.; Keohane, Carol; McSweeney, Maireade E.; Chung, Erica Y.; Yoon, Catherine S.; Lipsitz, Stuart R.; Wassner, Ari J.; Harper, Marvin B.; Landrigan, Christopher P. | 2013 | JAMA |
| 40 | Improving Patient Safety and Satisfaction With Standardized Bedside Handoff and Walking Rounds | Taylor, J. S. | 2015 | Clinical journal of oncology nursing |
| 41 | Physician Transition of Care: Benefits of I-PASS and an Electronic Handoff System in a Community Pediatric Residency Program | Walia, Jasmine; Qayumi, Zainab; Khawar, Nayaab; Dygulska, Beata; Bialik, Ilya; Salafia, Carolyn; Narula, Pramod | 2016 | Academic pediatrics |
| 42 | A multimodal intervention improves postanesthesia care unit handovers | Weinger, M. B.; Slagle, J. M.; Kuntz, A. H.; Schildcrout, J. S.; Banerjee, A.; Mercaldo, N. D.; Bills, J. L.; Wallston, K. A.; Speroff, T.; Patterson, E. S.; France, D. J. | 2015 | Anesthesia and Analgesia |
| 43 | Lost in relocation: longitudinal surveys evaluating the effectiveness of ICU to ward handover after the introduction of an electronic patient record | Westaway, S.; Webber, T.; Gluck, S.; Sundararajan, K. | 2022 | Hospital practice (1995) |
| 44 | Understanding how clinical judgement and communicative practices interact with the use of an electronic clinical handover system | Yee, K. C.; Wong, M. C.; Turner, P. | 2013 | Studies in Health Technology and Informatics |
| 45 | Quality and efficiency of a standardized e-handover system for pediatric nursing: A prospective interventional study | Zhou, Jiali; Zhang, Fen; Wang, Hansong; Yin, Yong; Wang, Qian; Yang, Lihua; Dong, Bin; Yuan, Jiajun; Liu, Shijian; Zhao, Liebin; Luo, Wenyi | 2022 | Journal of nursing management |
| 46 | Improving handoff with the implementation of I-PASS at a tertiary oncology hospital | Franco Vega, M. C.; Ait Aiss, M.; Smith, M.; George, M.; Day, L.; Mbadugha, A.; Niangar, Z.; Bodurka, D. | 2023 | BMJ Open Quality |
| 47 | Improving Communication Between ICU Nurses and Anesthesia Providers Using a Standardized Handoff Protocol | Bell, Erica; Benefield, Daniel; Vollenweider, Aaron; Wilson, Karissa; Warren, Laqueatrece Lashon; Aroke, Edwin N. | 2023 | Journal of perianesthesia nursing : official journal of the American Society of PeriAnesthesia Nurses |
| 48 | Improving the handover of complex trauma patients by implementing a standardised process | O'Neill, Kylie; Powell, Madeleine; Lovell, Tania; Brown, Duncan; Walsham, James; Calleja, Pauline; Nielsen, Sue; Mitchell, Marion | 2023 | Australian critical care : official journal of the Confederation of Australian Critical Care Nurses |
| 49 | Implementation of ED I-PASS as a Standardized Handoff Tool in the Pediatric Emergency Department | Yanni, Evan; Calaman, Sharon; Wiener, Ethan; Fine, Jeffrey S.; Sagalowsky, Selin T. | 2023 | Journal for healthcare quality : official publication of the National Association for Healthcare Quality |
| 50 | An evidence synthesis on perioperative Handoffs: A call for balanced sociotechnical solutions | Abraham, J.; Duffy, C.; Kandasamy, M.; France, D.; Greilich, P. | 2023 | Int. J. Med. Informatics |
| 51 | DESIGN AND IMPLEMENTATION OF A STRUCTURED APPLICATION-BASED INTRAOPERATIVE HANDOFF TOOL FOR ANESTHESIA CARE TEAMS: A QUALITY IMPROVEMENT APPROACH | Lai, Y. H.; Gui, J. L.; Arif, A.; Okorozo, A.; Van Patten, D. | 2023 | Middle East J. Anesthiol. |
| 52 | Machine learning to operationalize team cognition: A case study of patient handoffs | Mayes, E.; Gehlbach, J. A.; Jeziorczak, P. M.; Wooldridge, A. R. | 2023 | Human Factors Healthc. |
| 53 | Perioperative Handoff Enhancement Opportunities Through Technology and Artificial Intelligence: A Narrative Review | Sparling, J. L.; Hong Mershon, B.; Abraham, J. | 2023 | Jt. Comm. J. Qual. Patient Saf. |
