# Supplementary material for: Technological Solutions to Improve Inpatient Handover in the Era of Artificial Intelligence: Scoping Review
Source: J Med Internet Res. 2025 Jul 31;27:e70358. doi: 10.2196/70358 (PMC12312997; doi:10.2196/70358)
Supplement: Multimedia Appendix 4 [file jmir-v27-e70358-s004.docx]

**List of common advantages and disadvantages afforded by technological solutions with reference link to Multimedia Appendix 2**

| **Advantages** | **Referring article #** |
| --- | --- |
| Improved completeness, accuracy, and consistency of critical information during transfer | #1, #2, #4, #5, #13, #15, #16, #17, #18, #19, #25, #33, #41, #43, #45, #46, #47, #48, #49, #53 |
| Decreased adverse events due to handover error | #4, #13, #18, #25, #26, #37, #38, #39 |
| Improved quality of communication and handoff* | #1, #17, #18, #25, #28, #30, #46 |
| Improved handoff preparation efficiency | #3, #17, #32, #38, #45, #53 |
| Improved user satisfaction | #9, #17, #19, #22 |
| Reduced handoff length | #9, #31, #33 |
| More likely to communicate with patient's family and other healthcare workers | #24, #27 |
| Greater awareness of patients’ condition | #25, #44 |
| Users felt more prepared for handoff | #24 |
| Avoided confidentiality breaches with paper printouts | #26 |
| Increased standardized handoff tool utilization | #46 |
| Less time required to understand the patients' condition | #46 |
| Cost-effective solution for ensuring patient safety | #5 |
| More convenient handoff reporting | #25 |
| **Disadvantages** | **Referring article #** |
| Limited generalizability and scalability due to the limited scope of the analysis (e.g., specialty-specific, provider-specific, small dataset) | #1, #2, #4, #11, #20, #36, #37, #39, #49, #52, #53 |
| Difficult to ensure consistent adoption | #6, #12, #17, #23, #24, #44 |
| Requires regular updating of information, which may take time and increase workload | #4, #5, #7, #13, #14 |
| Requires user training, which may pose a steep learning curve for users | #14, #16, #22, #46, #53 |
| Limited accessibility for the handover tool due to limited infrastructure | #13, #14, #20, #24, #53 |
| Limitations in programming prevent the inclusion of necessary elements | #2, #20, #48 |
| Limited opportunities for clarification and requests for additional information | #6, #17, #47 |
| Prepopulation of the handover sheet means errors can be reproduced | #26, #33, #38 |
| Not completely automated and had to be manually inputted by staff | #2, #13 |
| No statistically significant improvement in patient outcomes with these tools | #8, #15 |
| Increase in average duration of verbal handovers | #37, 41 |
| Issues with compliance with data privacy on personal devices | #53 |

*as defined by the authors
